# Chlorambucil and lomustine (CL56) in absolute hormone refractory prostate cancer: re-induction of endocrine sensitivity an unexpected finding

**DOI:** 10.1038/sj.bjc.6602263

**Published:** 2004-11-30

**Authors:** J Shamash, G Dancey, C Barlow, P Wilson, W Ansell, R T D Oliver

**Affiliations:** 1 Department of Medical Oncology, St Bartholomew's Hospital, London EC1A 7BE, UK

**Keywords:** prostate cancer, hormone resistant, chemotherapy

## Abstract

The management of androgen independent prostate cancer is increasingly disputed. Diethylstilbestrol and steroids have useful second-line activity in its management. The value of chemotherapy still remains contentious. This paper reports a phase 2 study of two orally active chemotherapy drugs in patients who are absolutely hormone refractory having failed primary androgen blockade and combined oestrogens and corticosteroids. In total, 37 patients who were biochemically castrate with absolute hormone refractory prostate cancer and performance status of 0–3 were enrolled. Therapy consisted of chlorambucil 1 mg kg^−1^ given as 6 mg a day until the total dose was reached and lomustine 2 mg kg^−1^ given every 56 days (CL56). During this time all hormone therapy was stopped. One patient normalised his PSA with a further two having a greater than 50% decline leading to an objective response rate of 10%. The median time to progression was 3.6 months with an overall survival of 7.1 months. The median survival of this group of patients from first becoming androgen independent was 23.5 months. Eight of 17 (47%) patients who were subsequently re-challenged with hormonal therapy following failure of chemotherapy had a further PSA reduction, three (17%) of which were >50%. The median progression-free interval for the eight patients was 4 months. In conclusion, CL56 has a low objective response rate in the management of absolute hormone refractory prostate cancer. Toxicity was mild. Re-induction of hormone sensitivity following failure of chemotherapy was an unexpected finding that requires further study.

The majority of patients with locally advanced or metastatic prostate cancer treated by continuous androgen deprivation using either maximum androgen blockade (MAB) or GnRH analogue with deferred antiandrogens and then antiandrogen withdrawal ultimately become hormone resistant after 1–2 years treatment. Once hormone resistant their median survival then falls to about 1 year (Tannock *et al*, 1996).

Following the publication of three phase III studies comparing mitoxantrone and prednisone to prednisone alone in androgen independent prostate cancer, which demonstrated an improvement in symptom control despite failure to improve survival, chemotherapy has become accepted as having a role at this point in the disease (Tannock *et al*, 1996). More recently, the presentation of two large randomised phase III trials comparing mitoxantrone and docetaxel showed a significant survival advantage in favour of docetaxel (Eisenberger *et al*, 2004; Petrylak *et al*, 2004).

Oestrogens such as fosfosterol (Orlando *et al*, 2000) and diethylstilbestrol may produce responses in androgen independent patients. In one study the combination of low-dose diethystilboestrol and hydrocortisone produced a PSA response in 76% (Farrugia *et al*, 2000). Preliminary data suggesting that the response was more durable with dexamethasone than cortisone (Bhattacharyya *et al*, 2003) has led to that combination becoming the standard of care in our unit with chemotherapy only being used after second-line endocrine treatment fails.

In an attempt to investigate the true response rate in patients who had failed primary androgen deprivation as well as both oestrogens and corticosteroids (during which GnRH analogue therapy was stopped). A phase II chemotherapy study was undertaken. Patients who were not biochemically castrate on steroids and oestrogens and had a rising PSA had GnRH therapy reinstituted and only when this failed to control their PSA were they eligible for chemotherapy. To increase patient acceptance two orally active chemotherapy agents, chlorambucil and lomustine, were selected. These were active drugs when tested in the early 1980s by less stringent criteria than used today and had response rates of 30–46% (Catane *et al*, 1978; Loening *et al*, 1981; Herr, 1982; Ruff *et al*, 1989). Although these earlier results are of use in suggesting some activity, the absolute percentages cannot be compared to the more recent data from the post-PSA era. As the combination had shown good activity with low toxicity in refractory lymphoma (Oza *et al*, 1993), we set out to study this combination in absolute hormone refractory prostate cancer. This paper reports these preliminary findings.

## PATIENTS AND METHODS

Patients were eligible for the study if they had histologically documented adenocarcinoma of the prostate with progression despite androgen deprivation followed by second-line hormonal therapy with steroids and oestrogens and were chemotherapy naïve.

On becoming androgen independent (as judged by rising PSA in the presence of castrate levels of testosterone (<1.5 nmol l^−1^)) patients were treated with diethylstilbestrol 1 mg day^−1^ combined with a steroid (either dexamethasone 2 mg day^−1^ or hydrocortisone 20 mg twice a day or prednisolone 20 mg day^−1^) without a GnRH analogue. Following documented biochemical or symptomatic progression, the serum testosterone was rechecked and if they were still biochemically castrate they were eligible for chemotherapy. If not, androgen deprivation was recommenced and only if there were no response to this would chemotherapy be offered. Patients had their GnRH therapy stopped at the start of chemotherapy.

Assessable disease consisted of soft tissue disease or a rising PSA. Patients were required to have an Eastern Cooperative Oncology Group status of 0–3. Required haematological parameters were platelets greater than 100 × 10^9^ l^−1^, granulocytes greater than 3 × 10^9^l^−1^ and haemoglobin greater than 90 g l^−1^ (transfusions were permitted). The study was approved by Local Research Ethics Committee (LREC) and all patients gave written informed consent prior to participation.

### Treatment and evaluation

Treatment consisted of chlorambucil at 1 mg kg^−1^ (given at 6 mg a day until the total dose reached) and lomustine at 2 mg kg^−1^ on day 1. The dose of lomustine was reduced to 1 mg kg^−1^ if radiotherapy to more than three sites had previously been given. Granisetron 1 mg orally day 1, dexamethasone 8 mg day 1 and 4 mg day^−1^ on days 2 and 3 as well as metoclopramide 20 mg 3 × /day for 3 days were prescribed as antiemetics. This cycle was repeated every 56 days (CL56). Patients were asked to complete quality of life forms both general and prostate specific. (EORTC – QLQ 30 with PR 25 prior to treatment and on day 1 on subsequent cycles). Once started on chemotherapy all hormone therapy was stopped, including if possible, a phased reduction of steroids. If pain worsened or the patient developed steroid deficiency symptoms, they remained on their original dose of steroids until it was clear whether they were responding when a further phased reduction was attempted.

Patients were evaluated for response at the end of cycle 1. If the disease was responding or stable, further cycles were given to a maximum of six cycles. Progression (PD) on PSA was defined as an increase of greater than 25% over nadir that was then confirmed with a second rising PSA. Those patients with soft tissue disease were assessed on CT scan. Repeat blood tests were carried out on days 1, 22 and 36 of each cycle, including a full blood count, urea and electrolytes, alkaline phosphatase and PSA.

The date of progression was defined as that of the first CT that showed new lesions or a 25% increase in previously measured disease, or showing a rise in PSA. PSA responses were evaluated using the guidelines from the prostate-specific antigen working group for PSA response in androgen independent disease (Bubley *et al*, 1999). In the first cycle if the PSA was rising but there were clinical signs of improvement then a second cycle was given. If it became clear that the PSA continued to rise in the second cycle then the date of progression was taken on the first confirmed rise in PSA. In some patients, this was 21 days into the first cycle. For those whose PSA rose in the first 8 weeks and then fell, the date of progression was the first confirmed rise in the second or subsequent cycle. A 50% fall in PSA maintained for 4 weeks was termed a partial response (PR). Patients whose PSA stabilised for more than 8 weeks, that is, did not rise by 25% or fall by greater than 50% were considered as stable disease. Normalisation of PSA (less than 4 ng ml^−1^) confirmed for 1 month was termed a complete response (CR).

### Dose modifications

Prior to each chemotherapy cycle, platelets greater than 80 × 10^9^ l^−1^ and white cell count greater than 3.0 × 10^9^ l^−1^ were needed. Patients with low counts were delayed for a week or until the blood count recovered. Failure to recover counts in 4 weeks meant the patient went off study.

If a delay was required, the doses of lomustine and chlorambucil were reduced by 25%. If the nadir platelet count was <20 × 10^9^ l^−1^, the dose of lomustine was reduced by 50% on subsequent cycles. If nadir platelet count was between 21 and 49 × 10^9^ l^−1^, the subsequent lomustine dose was reduced by 25%.

Patients who had received prior treatment with strontium or previous radiotherapy to three or more sites received a reduced dose of lomustine of 1 mg kg^−1^. If the nadir platelet count was greater than 50 × 10^9^, the lomustine dose was increased to 2 mg kg^−1^ on subsequent cycles.

### Statistical design and analyses

The log-rank test was used to look at which variables were significant on univariate analysis for overall survival and progression-free survival. The Wilcoxon matched pairs signed rank sum test was used to look at changes in Quality of Life scores from baseline to 8 weeks. Fishers exact test was used when the numbers expected were less than 5.

## RESULTS

### Patient characteristics (Table 1)

Between the 3 November 2000 and the 20 September 2002, 37 patients were recruited into this study, their median performance status was 1 (range 0–3). A total of 89% were symptomatic and 19% had extensive visceral disease (liver or lung metastases). Only 48% had normalised their PSA with androgen deprivation despite the fact that 49% had received initial MAB. The median time to androgen independence for the group was 23.5 months and the median time between androgen independence and receiving chemotherapy was 14 months.

### Toxicity (see Table 2)

A median of two cycles of therapy was administered. No grade 4 toxicity was seen. The most significant problems were malaise (grade 3 in 14%), and constipation (grade 3 in 12%), which reflects in part the high frequency of opioid analgesia in this group of advanced end stage patients. Haematological toxicity was mild with only 4% having grade 3 leukopenia and 11% grade 3 thrombocytopenia.

### Treatment outcomes

The overall results are summarised in Table 3. Biochemical response rate to CL56 was 10% (one normalisation of PSA and two with a greater than 50% response). An additional 46% achieved stabilisation of their disease beyond 8 weeks and overall 54% had a symptomatic response. The median time to progression was 3.6 months (95% CI 2.3–4.9) (CR/PR 4.4 months, SD 3.7 months, PD 0.9 months) with an overall survival of 7.1 months (CR/PR 8.8 months, SD 10.9 months, PD 4.8 months). The median survival from becoming androgen independent was 23.5 months (95% CI 17.0–29.8).

Eight out of 17 patients with SD had an initial rise in PSA in the first 3–8 weeks (median rise 99% range 8–794%) followed by plateau and then a fall 2 weeks later (median fall 29% from the peak, range 7–63%). In three of eight, these declines lasted more than 24 weeks though in two of the three, the minimum was still higher than the pretreatment level.

Those who had SD at 8 weeks had a different subsequent pattern of symptom response and PSA change than those who progressed.

The PSA levels (median 198 ng ml^−1^ for SD and 206 ng ml^−1^ for PD) and PSA doubling time prior to the start of chemotherapy (median 80 days for SD and 78 days for PD/NE) were very similar for the SD group and the PD/NE group. Those who had objective biochemical responses had lower PSA levels (median 80 ng ml^−1^) and doubling time (median 46 days) at the start of chemotherapy but numbers were small. However after treatment, there was a marked difference in post-treatment PSA doubling time between the SD and PD groups (217 *vs* 29 days).

Following progression on CL56, endocrine treatment was restarted in 17 patients (Table 4). Two orchidectomised patients failed to respond to addition of antiandrogens, though the only one of these to be retreated with stilbestrol and dexamethasone after failed antiandrogens responded a second time. In total, 13 patients received GnRH analogue for a second time. There were six PSA falls, two of which were greater than 50%. Six of the 13 patients subsequently received diethylstilbestrol and dexamethasone after further progression on GnRH and two responded. There was in addition one who received diethylstilbestrol and dexamethasone immediately after progression on CL56, and responded with a fall in PSA of 45%. The median time from CL56 to starting hormone therapy was greater in those who subsequently responded to hormones again although this did not reach statistical significance (8.0 *vs* 3.8 months, *P*=0.085).

Hormone profiles (LH, FSH and testosterone) were repeated in nine patients before secondary endocrine treatment was restarted. Two were found not to be castrate and both responded to subsequent treatment (15 and 55% falls in PSA falls, respectively). Seven were castrate and two responded.

A total of 17 patients, who completed at least two courses of treatment, returned quality of life questionnaires. Eight patients showed some improvement (median improvement +1.5 (quality of life raw score)). In four patients there was no change. In five, quality of life declined (median decline 2.0). There was no clear relationship between Quality of Life changes and tumour response.

### Prognostic factors

In terms of overall survival and time to progression, the following were analysed: Gleason score, normalisation of PSA with prior androgen deprivation, raised alkaline phosphatase, performance status, PSA level, time to androgen independence, age, previous steroid use (dexamethasone vs alternative steroid), period of time of androgen independence prior to chemotherapy.

Of these, on univariate analyses only raised alkaline phosphatase (5.5 months *vs* 8.8, *P*=0.048), PSA above median (>185 ng ml^−1^) (5.5 *vs* 8.8 months *P*=0.024) and time to androgen independence of <1 year prior to chemotherapy (5.5 *vs* >6.4 months *P*=0.03) were significant for overall survival. For progression-free survival, only prior dexamethasone *vs* another steroid (4.3 *vs* 2 months *P*=0.013) and time to androgen independence of >2 years (4.2 *vs* 2.4 months *P*=0.049) were significant.

## DISCUSSION

The objective response rate to the combination of chlorambucil and lomustine (CL56) was modest (10%) and at first sight not very encouraging.

The study was undertaken with these two drugs because the response rate when the two drugs were used together in chemorefractory lymphoma was higher than when used as single agents (Oza *et al*, 1993).

There was a suggestion that patients with a lower PSA might be more likely to respond as all responses occurred in patients with PSA <110 ng ml^−1^ (three out of 14 responses, PSA ⩾110 ng ml^−1^ zero out of 18 responses (*P*=0.04 two-tailed Fisher's exact test).

Patients who had stable disease at 8 weeks had a change in their PSA velocity with a substantial reduction in doubling time during chemotherapy. With the use of PSA velocity as a surrogate marker being increasingly accepted, such findings may assume greater significance (Carter *et al*, 2003; D'Amico *et al*, 2003; Rini *et al*, 2003). The lower response rate in this study particularly in comparison to newer data from taxanes based studies (Eisenberger *et al*, 2004; Petrylak *et al*, 2004) may in part be explained by the fact that the patients in this study were resistant to corticosteroids and oestrogens as well as to androgen deprivation.

The action of steroids in androgen independent prostate cancer prior to chemotherapy appears more complicated than first thought. The rationale for their use and dosing in this setting was to suppress adrenal production of androgens. It appears that other mechanisms may be important as different steroids have different PSA response rates. In the recent preliminary analysis of a randomised trial comparing dexamethasone alone to dexamethasone and diethylstilbestrol (Shamash *et al*, 2004), 51% had a PSA response to dexamethasone. This is higher than the PSA response to hydrocortisone – 21% (Dawson *et al*, 1995; Kantoff *et al*, 1999), or prednisone – 17% (Dowling *et al*, 2001; Berry *et al*, 2002). One explanation for this may be that receptors other than the androgen receptor may be more important than previously appreciated in determining prostate cancer responses. This raises the question as to whether the hormonal responses postchemotherapy represent separate clones being suppressed or whether the chemotherapy is modulating the plasticity of the receptor profile on the tumour cell. It is also possible that the routine use of MAB in some studies would have removed any androgen sensitive clones that had survived GnRH monotherapy and it is these clones that were responsible for the re-response to GnRH therapy following the cessation of chemotherapy in this study.

A criticism of this study is that patients were not androgen suppressed during chemotherapy. It could be that although most remained biochemically castrate that androgen level could have been kept lower by maintaining androgen deprivation and even a small rise within the castrate range was responsible for the growth of androgen dependent clone, which then responded to the reinduction of androgen deprivation following the failure of chemotherapy. We do not believe this to be the dominant explanation. There are anecdotal reports of similar secondary responses being seen in breast cancer patients progressing on chemotherapy (Ragaz, 2001), and they provide additional arguments for the use of sequential hormonal and then chemotherapy rather than the use of both modalities consecutively.

In conclusion, this study has demonstrated that use of a simple oral chemotherapy regimen repeated every 8 weeks sequentially following the failure of oestrogens and steroids in the management of androgen independent prostate cancer produces evidence of antitumour activity in nearly 2/3rds of such end stage patients based on slowing of tumour doubling time despite a low PSA response rate. It is well tolerated and the survival in this cohort compares favourably with that seen in the current more toxic taxane/estramustine based combinations. There is an urgent need to prospectively analyse secondary hormone responses following chemotherapy so that their frequency and duration may be more accurately determined.

## Figures and Tables

**Table 1:** Pretreatment patient characteristics

	**Patient characteristics**
Number of patients	37
Median age (range)	71.4 years (57.6–83.9)
	
*Disease extent*	
Localised	1
Bone mets only	13
Bone+nodal disease	16
Bone+liver/lung	7
	
*Performance score*	
0	2
1	18
2	11
3	5
Missing	1
	
*Symptomatic*	
No	3
Yes	33
Missing	1
	
*Primary therapy*	
GnRH	18
MAB	13
Orchidectomy	3
Antiandrogen monotherapy	3
	
*PSA levels at start CL56*	
Median and (range)	183.5 (11.3–1560)
	
*Alkaline phosphatase at start of CL56*	
⩽120	18
>120	19
	
*Gleason score*	
8–10	6
5–7	17
⩽4	3
Missing	11
	
*PSA normalised with prior androgen deprivation*	
No	16
Yes	15
Missing	6
	
*Median time to androgen independence (AI)*	25 months (range 4.4–136.7)
Number AI<2 years	17
Number AI>2 years	20
	
Median time AI to start study	14.3 months (range 1.8–46.2)
Number AI to start study<1 year	17
Number AI to start study >1 year	20

**Table 2:** WHO toxicity grade per cycle (in percentages)

	**Grade 1–2 (%)**	**Grade 3 (%)**	**Grade 4 (%)**
Infection	4	0	0
Malaise	29	14	0
Taste	4	0	0
Nausea	14	2	0
Vomiting	2	0	0
Constipation	14	12	0
Diarrhoea	0	0	0
Stomatitis	0	0	0
Anorexia	17	8	0
Alopecia	0	0	0
Dyspnoea	10	0	0
Neuropathy	0	0	0
			
Hb	15	4	0
Wbc	20	4	0
Platelets	13	11	0

**Table 3:** Response and survival following CL56 and subsequent response to further hormone therapy

Median no. of cycles:	2 (range 1–6)
*Response to CL56*	
CR	1
PR	2
SD	17
PD	12
NE	5
	
*Response rate*	
	3/32 (10%)
	
*Stabilisation rate (SD or better)*	
	20/32 (63%)
	
*Symptomatic improvement (24 evaluable)*	
No	13
Yes	11
	
*Alk Phos response by week 8 (14 evaluable)*	
No	11
Yes	3
Median survival	7.1 months (95% CI 6.2–8.0)
Median time to progression	3.6 months (95% CI 2.3–4.9)
PF at 6 months	5/37
Median survival following AI	23.5 months (95% CI 17.2–29.8)
	
*Post-CL56 hormones*	
Yes	17
	
*Response to post-CL56 hormone therapy*	
Yes	9
>50%	3

**Table 4:** Post-CL56 hormone characteristics and subsequent response in 17 patients

**Post-CL56 treatment**									
**Patient no.**	**Castrate**	**GnRH analogue**	**MAB**	**% PSA decline^a^**	**Duration (months)**	**Stilb/steroid**	**% PSA decline^a^**	**Duration (months)**	**Time from CL56 to postchemotherapy hormone treatment (months)**
1	Yes	Yes	Yes	PD		No	—	—	3.6
2^b^	Yes	No	Yes	PD		Yes	90%	11	19.0
3	Yes	Yes	Yes	PD		No	—	—	3.2
4	Yes	Yes	No	10%	2.5	Yes	20%	4	8.0
5	Yes	Yes	No	PD	—	Yes	PD	—	5.5
6	Unknown	No	No	—	—	Yes	45%	4	4.6
7	Unknown	Yes	Yes	PD	—	Yes	PD	—	4.0
8^b^	Yes	No	Yes	PD	—	—	—	—	8.2
9	No	Yes	No	55%	6	Yes	PD	—	9.1
10	Unknown	Yes	Yes	97%	6.0+	No	—	—	16.1
11	Unknown	Yes	No	45%	2	No	—	—	3.8
12	Yes	Yes	No	7%	3	No	—	—	7.2
13	Yes	Yes	No	PD	—	—	—	—	2.5
14	Yes	Yes	No	PD	—	—	—	—	3.8
15	Unknown	Yes	Yes	30%	3	Yes	PD	—	
16	Yes	No	Yes	PD	—	—	—	—	3.7
17	No	Yes	No	PD	—	Yes	15%	2	3.8

MAB=maximum androgen blockade.

Stilb/steroid=diethylstilbestrol and steroid.

a From the start of hormone therapy.

b Patient had orchidectomy.
